# A designer hyper interleukin 11 (H11) is a biologically active cytokine

**DOI:** 10.1186/1472-6750-12-8

**Published:** 2012-03-21

**Authors:** Hanna Dams-Kozlowska, Katarzyna Gryska, Eliza Kwiatkowska-Borowczyk, Dariusz Izycki, Stefan Rose-John, Andrzej Mackiewicz

**Affiliations:** 1 Department of Cancer Diagnostics and Immunology, Greater Poland Cancer Centre, 15 Garbary St, 61-866 Poznan, Poland; 2 Chair of Medical Biotechnology, Poznan University of Medical Sciences, Poznan, Poland; 3 Department of Biochemistry, Christian-Albrechts-University, Kiel, Germany

**Keywords:** IL-11, sIL-11Rα, Hyper-IL11, Fusion protein, gp130 targeting, Hypercytokine

## Abstract

**Background:**

Interleukin 11 (IL-11) is a pleiotropic cytokine with anti-apoptotic, anti-inflammatory and hematopoietic potential. The IL-11 activity is determined by the expression of the IL-11R receptor alpha (IL-11Rα) and the signal transducing subunit β (gp130) on the cell membrane. A recombinant soluble form of the IL-11Rα (sIL-11Rα) in combination with IL-11 acts as an agonist on cells expressing the gp130 molecule. We constructed a designer cytokine Hyper IL-11 (H11), which is exclusively composed of naturally existing components. It contains the full length sIL-11Rα connected with the mature IL-11 protein using their natural sequences only. Such a construct has two major advantages: (i) its components are as close as possible to the natural forms of both proteins and (ii) it lacks an artificial linker what should avoid induction of antibody production.

**Results:**

The H11 construct was generated, the protein was produced in a baculovirus expression system and was then purified by using ion exchange chromatography. The H11 protein displayed activity in three independent bioassays, (i) it induced acute phase proteins production in HepG2 cells expressing IL-11, IL-11Rα and gp130, (ii) it stimulated the proliferation of B9 cells (cells expressing IL-11Rα and gp130) and (iii) proliferation of Baf/3-gp130 cells (cells not expressing IL-11 and IL-11Rα but gp130). Moreover, the preliminary data indicated that H11 was functionally distinct from Hyper-IL-6, a molecule which utilizes the same homodimer of signal transducing receptor (gp130).

**Conclusions:**

The biologically active H11 may be potentially useful for treatment of thrombocytopenia, infertility, multiple sclerosis, cardiovascular diseases or inflammatory disorders.

## Background

IL-11 is a pleiotropic cytokine, which exhibits multiple biological activities which are determined by expression of IL-11Rα and gp130 on the cell membrane [1]. Originally it was identified in 1990 as a molecule promoting growth of the IL-6-dependent mouse plasmacytoma cell line B9 [2]. It has been demonstrated later, that IL-11 exhibits multiple effects not only on hematopoietic system, but it also acts on various cell types of the liver, gastrointestinal tract, lung, heart, central nervous system, bone, joint and immune system [1]. IL-11 acts synergistically with other growth factors in the process of hematopotic cells differentiation including progenitor cells, and on megakaryocytopoiesis, thrombopoiesis, erythropoiesis and myelopoiesis [1]. Moreover, IL-11 displays anti-melanoma activity when used as molecular adjuvant in the therapeutic whole cell melanoma vaccine formulation [3].

IL-11 together with IL-6, IL-27, Leukemia Inhibitory Factor (LIF), Oncostatin M (OSM), Ciliary Neurotrophic Factor (CNTF), Cardiotrophin 1 (CT-1), cardiotrophin-like cytokine (CLC) and neuropoietin (NP) belongs to the family of hemopoietic cytokines (named IL-6-type or gp130 cytokines), which share structural similarity and a common receptor subunit (gp130) [4,5]. Some of the IL-6-type cytokines require a specific (unique) receptor complex, however, always one or two subunits of a common transmembrane transducer receptor gp130 is required. IL-6 and IL-11 engage a homodimer of gp130. Other IL-6-type cytokines like LIF, CT-1, CNTF, NP, CLC need LIF receptor (LIFR) and gp130. OSM binds first to gp130 and then with either LIFR or OSMR. IL-27 forms signaling complex with gp130 and WSX-1 (IL-27R). Moreover, some of the IL-6 type interleukins first bind to a specific receptor alpha and then engage signal transducer subunits [6]. Cytokines utilizing gp130 molecule induce signaling via the Janus Kinase/Signal Transducer and Activator of Transcription (JAK/STAT) pathway and also the Mitogen-Activated Protein Kinase (MAPK) cascade [7].

Specifically, the IL-11 receptor complex is formed via three separate events. First, IL-11 binds with low affinity to membrane specific receptor *α*. Next, IL-11/IL-11Rα heterodimer binds with high affinity to receptor gp130 forming heterotrimer. At the last stage, the heterotrimers associate forming a hexameric complex that elicits the biological response. The stoichiometry of the high affinity IL-11 receptor complex has been determined *in vitro *as a hexamer consisting of two IL-11 molecules, two IL-11Rα chains and a homodimer of two gp130 molecules [8].

The gp130 protein was found on all human cell types so far studied, however the expression of other IL-6-type receptor subunits is limited. Cells that express the proper subunit will be sensitive to the respective cytokine. Moreover, soluble forms of the alpha receptors lacking the transmembrane and cytoplasmic domains were found [7]. Soluble forms of cytokine receptors can be produced by limited proteolysis of the membrane-bound receptor or by translation of alternatively spliced mRNA [9]. Cells that do not express receptor α can be sensitive to the complex of cytokine/soluble cytokine receptor α. Recombinant soluble IL-11Rα (sIL-11Rα) can bind IL-11 and then the comples attracts gp130 leading to signal transduction [10]. The complex of IL-11/sIL-11Rα can activate cells bearing both IL-11Rα and gp130 subunits or gp130 only. The recombinant sIL-11Rα acts *in vitro *as IL-11 agonist, although antagonizing activity was also observed [10-12].

In order to increase and modify the potential bioactivity of some cytokines, the idea of linking of two soluble, naturally existing components was postulated. The fusion proteins are expected to be more stable and are needed at a lower effective dose. Examples derived from IL-6-type cytokines include fusion proteins consisting of different fragments of alpha receptors and their cognate cytokines like IL-6/sIL-6R (Hyper IL-6), IL-11/sIL-11Rα (IL-11/R-FP) and CNTF/sCNTFR (Hyper-CNTF) [13-15]. Here, we constructed a novel cytokine Hyper IL-11 (H11), which is composed of naturally existing components only. It contains the full length soluble IL-11Rα connected with IL-11. Since the natural sequence of C-terminus of IL-11Rα and N-terminus of IL-11 were used, we could refrain from using artificial linker sequences for the connection of both components.

## Results

### Construction of H11

**Figure 1:**
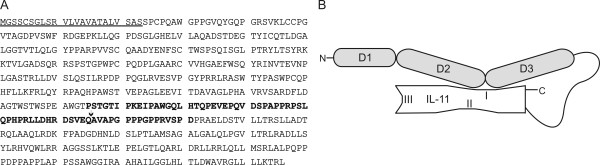
**(A) Amino acid (AA) sequence of the fusion protein H11 and (B) Schematic representation of tertiary structure of the fusion complex**. (A) The N-terminal signal peptide is indicated by thick underline. The sequences of C-terminus of IL-11Rα and N-terminus of IL-11, which form natural linker are boldface. The arrow indicates the fusion site. (B) The Ig-like region (D1 domain) and cytokine binding domains (D2 and D3) of IL-11Rα are indicated. The three receptor binding sites of IL-11 are shown. IL-11 binds the IL-11Rα though site I, while site II and III are occupied by two gp130 molecules.

Our new designer cytokine Hyper IL-11 (H11) is composed exclusively of naturally existing components. It contains the full length soluble IL-11Rα connected with IL-11. The cDNA fragment of s IL-11Rα (position 1-1095) and the cDNA of IL-11 (position 55-600) were amplified by PCR. The forward primer contained an additional recognition sequence for the restriction enzyme *Sal*I. Moreover, ACC nucleotides in front of ATG and substitution A for G just after the translational start codon ATG were introduced in order to provide the Kozak consensus sequence and a restriction site *Nco*I, respectively. The reverse primer for amplification of IL-11Rα contained an additional 5' overlapping sequence on IL-11 at position 86 where a natural restriction site *Xho*I occurs. Moreover, the amplified fragment omitted the leader sequence of IL-11 with exception of natural sequence coding the last three amino acids (AVA) of the leader sequence which are now at the N termini of the IL-11 component. The restriction site *Xho*I was used to connect both components. The membrane proximal region of the IL-11Rα (51 amino acids) and the 16 N-terminal residues of IL-11 are not helical and presumably flexible enabling the proper assembly of the complex. The sequence and schematic representation of the fusion complex are shown in Figure 1. Moreover, the details of the H11 construction have been described in Patent No WO2005113591 [16].

### Production and purification of H11 protein

**Figure 2:**
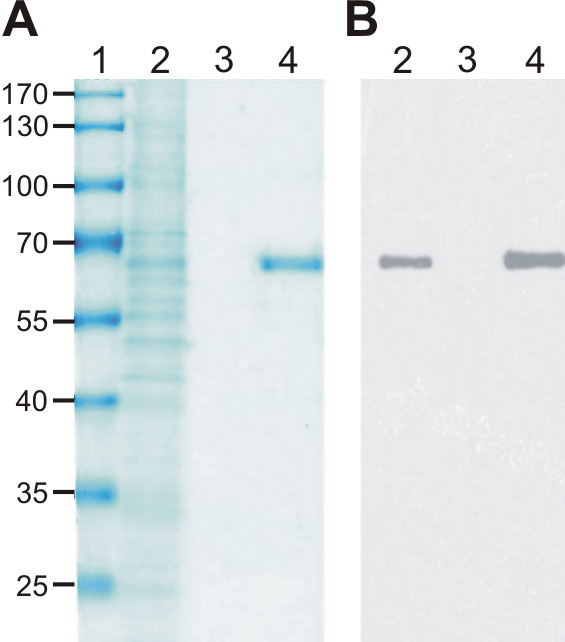
**Purification of H11-SDS-PAGE (A) and Western Blot (B) analysis**. The proteins were visualized with Roti-Blue stain and for detection H11 the anti- IL-11Rα antibody (Santa Cruz Biotechnology, Santa Cruz, CA) was used. Lane 1- molecular weight marker PageRuler (Fermentas), lane 2-supernatant from infected High-Five BTI-TN-5B1-4 cells, 3-supernatant diluted with 20 mM 1.3 DAP (1:8), 4-eluted H11.

The production of recombinant H11 protein was carried out in a Baculovirus expression system. High-Five BTI-TN-5B1-4 insect cells were used, which were transduced at a density of 1x10^6 ^cells/ml using MOI 5. The H11 production was carried out for 48 h in Express Five SFM medium. The purification of fusion protein was carried out by ion exchange chromatography. Purified H11 from the Q Sepharose XL bed was eluted at 50 mM NaCl/20 mM 1,3 diaminopropane buffer pH 10.5. The quality and quantity of the fusion protein was determinated by SDS-PAGE gel electrophoresis and Western blot analysis (Figure 2). The intensive band, which migrated in SDS-PAGE below 66 kDa BSA (data not shown), was recognized by anti- IL-11Rα antibody which binds H11. The observed molecular weight of H11 was slightly above the calculated one (58.8 kDa) what might be due to the N-glycosylation. The N-glycosylation of IL-11Rα was described before [13]. Although, H11 produced in insect cells may differ in pattern of glycosylation when compared with one produced in mammalian cells [17]. the glycosylation was shown not to be critical for ligand gp130 interaction [18]. The average yield of purified H11 was 646 μg from 1 L of cell supernatant.

### Activity of H11

The purified recombinant H11 was tested for biological activity in three different *in vitro *bio-assays: human hepatoma cell line HepG2-assay, murine B9 (hybridoma cell line) bio-assay, and murine Ba/F3-gp130 bio-assay.

**Figure 3:**
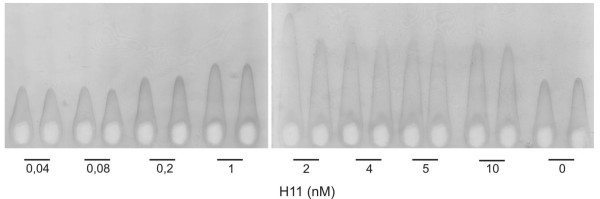
**Rocket immunoelectrophoresis of supernatants from HepG2 cells**. HepG2 cells were stimulated with recombinant H11 at various concentrations. After 48 h the collected medium was analyzed by rocket immunoelectrophoresis for presence of α_1_-antitrypsin.

*Stimulation of α_1 _antitrypsin in HepG2 cells by H11*. HepG2 cells secrete endogenous IL-11, which makes them unresponsive to exogenous IL-11. However, HepG2 insensibility to IL-11 can be restored by the addition of s IL-11Rα [10]. Incubation of HepG2 cells with H11 stimulated in a dose dependent manner the expression and secretion of a read-out plasma protein-α_1 _antitrypsin as measured by rocket immunoelectrophoresis (Figure 3).

**Figure 4:**
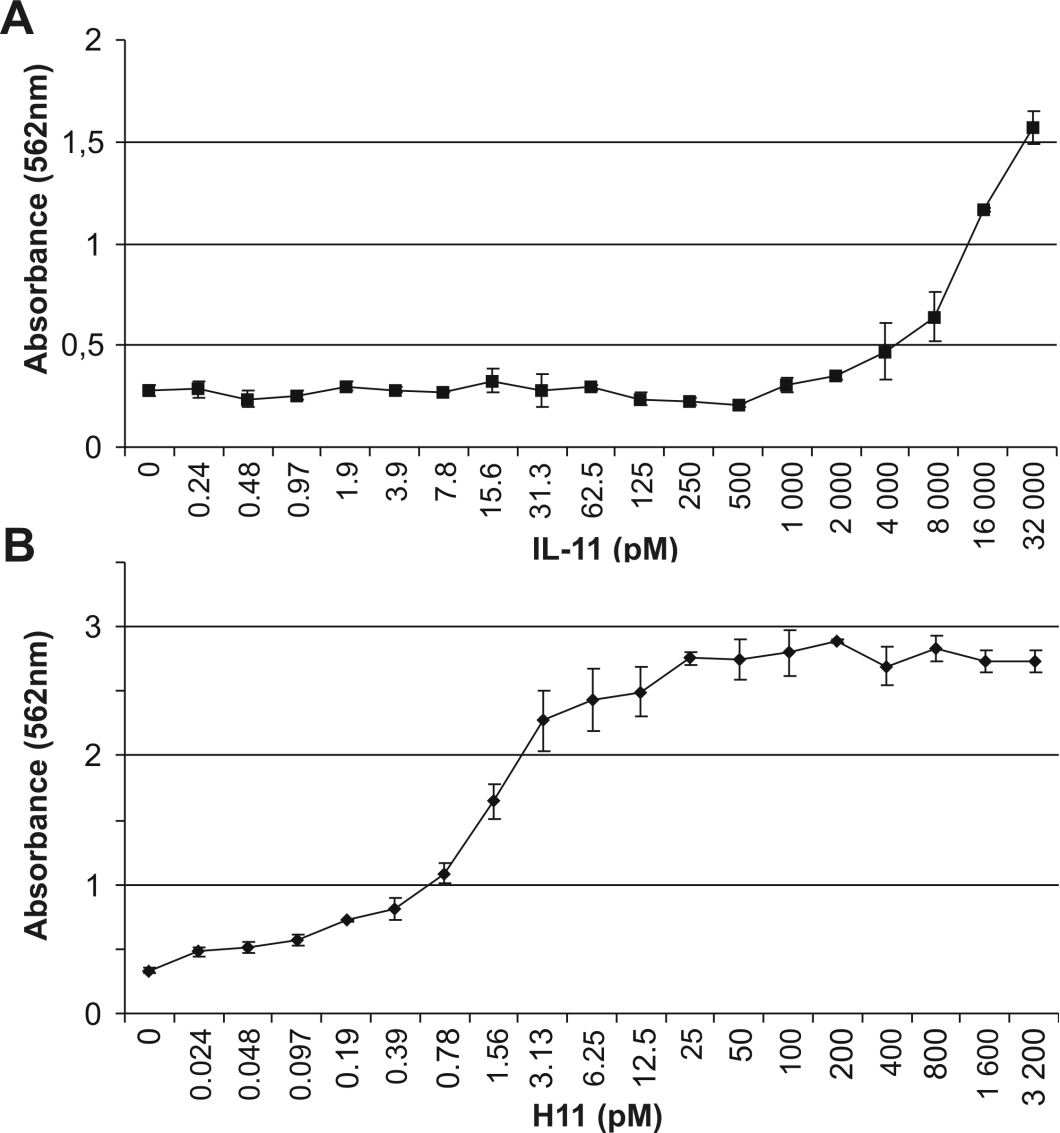
**The proliferation assay of B9 cells**. B9 cells (1 × 10^4 ^cells per well) were tested for proliferation at various concentrations of (A) IL-11 and (B) H11. The medium without stimulator was used as the negative control. The proliferation of the cells was measured after 3 days using colorimetric assay based on MTT reagent.

*Proliferation of B9 cells*. B9 cells express IL-11Rα and gp130 receptors, making them responsive to IL-11 [19]. Stimulation of B9 cells with IL-11 and H11 led to induction of their proliferation (Figure 4). To obtain comparable levels of B9 proliferation, H11 was applied at a 10-fold lower molar concentration than IL-11. The highest proliferation of B9 cells was achieved with 32000 pM IL-11. Since no saturation was reached at this concentration, we are not sure whether application of higher IL-11 concentration would increase B9 proliferation further. Application of IL-11 at higher dose was impossible due to the technical reasons. When B9 cells were stimulated with H11, a concentration of 6.25 pM was sufficient for maximal proliferation. The difference between IL-11 and H11 activity was probably due to the low expression of the IL-11 receptor. Indeed, H11 composed of active IL-11 and sIL-11Rα induced much stronger proliferation of B9 cells.

**Figure 5:**
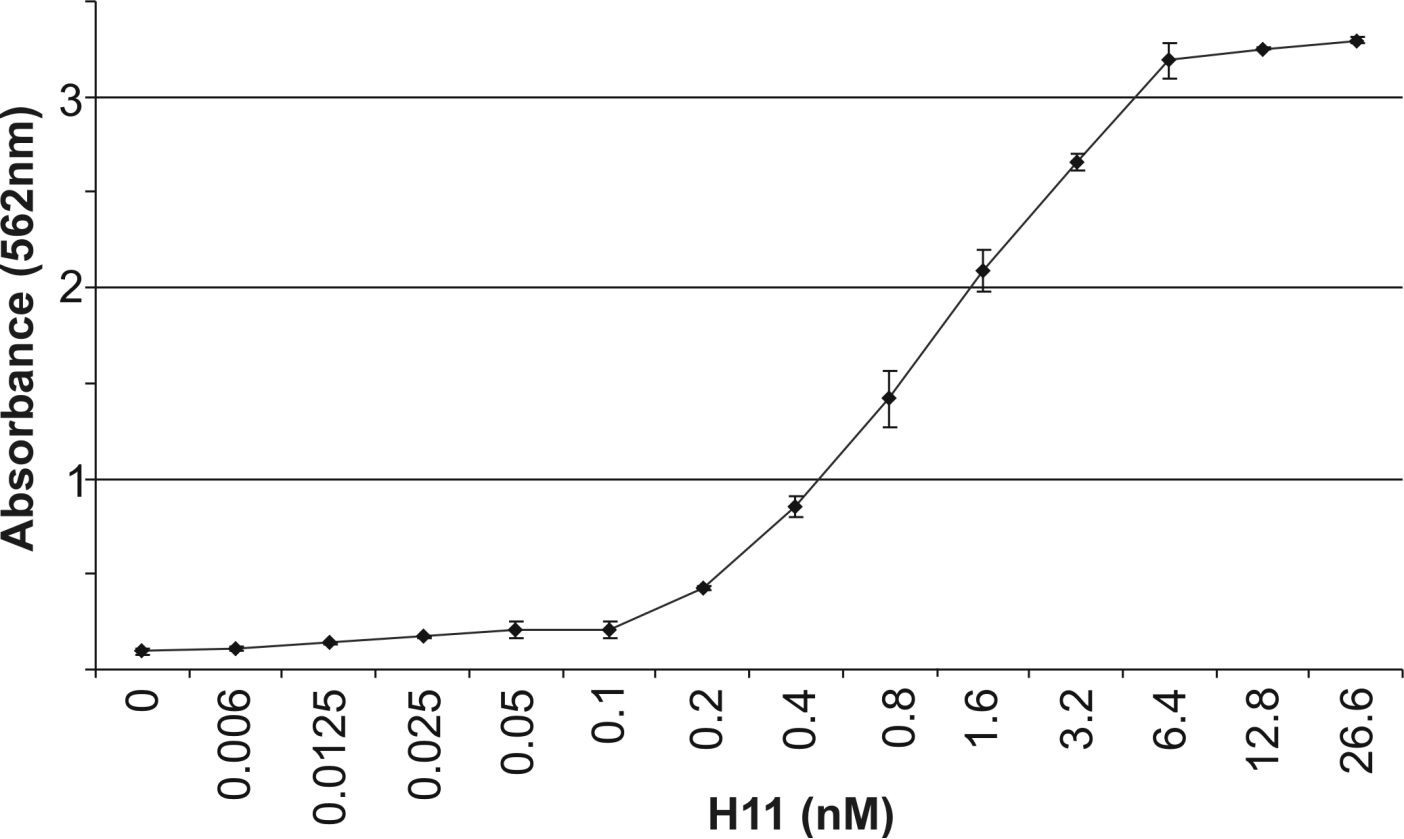
**The proliferation assay of Ba/F3-gp130 cells**. Ba/F3-gp130 (2 × 10^4 ^cells per well) cells were induced by H11 at various concentrations. The medium without stimulator was used as the negative control. The proliferation of the cells was measured after 3 days using colorimetric assay based on MTT reagent.

*Proliferation of Ba/F3-gp130 cells*. The unique feature of Ba/F3 cells (pro-B lymphocyte cell line) is the lack of membrane gp130 molecules, which makes them unresponsive to IL-6 type cytokines. However, stable transfection of Ba/F3 cells with gp130 cDNA made them responsive to the combination of IL-6 type cytokine and its α receptor [13,14]. Stimulation of Ba/F3-gp130 cells with recombinant H11 led to their propagation, as measured using MTT test (Figure 5). Ba/F3-gp130 cells did not proliferate in medium without stimuli however, H11 stimulated these cells in a at dose dependent manner. The Ba/F3-gp130 assay proved that the new designer cytokine H11 was acting as a complex.

### Signal transduction mediated by H11

**Figure 6:**
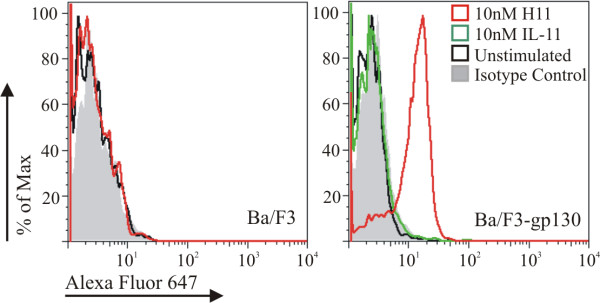
**Phosphorylation of transcription factor STAT 3 analyzed by flow cytometry**. Ba/F3 and Ba/F3-gp130 cells were stimulated with control medium (unstimulated), hIL-11 or H11 and phosphorylation of STAT3 was measured using anti- STAT3P antibody.

Signal transduction of IL-11 and related cytokines via the common signal transducer gp130 is mediated by the activation Jak/STAT and Ras/MAPK signaling pathways [9]. Active Jaks mediate phosphorylation of tyrosine residues of the receptors and subsequent recruitment of STAT3/1 and SHP-2. In order to show that STAT3 activation was effected by H11, Ba/F3-gp130 cells were stimulated with the hypercytokine and the phosphorylated forms of STAT3 (pSTAT3) were detected using flow cytometry. As indicated in Figure 6, contrary to IL-11, the stimulation with H11 led to activation of STAT 3 molecule, indicating that H11 activity was not due to an altered mechanism.

### Differentiation study of hematopoietic Lin-CD34+ cells

**Figure 7:**
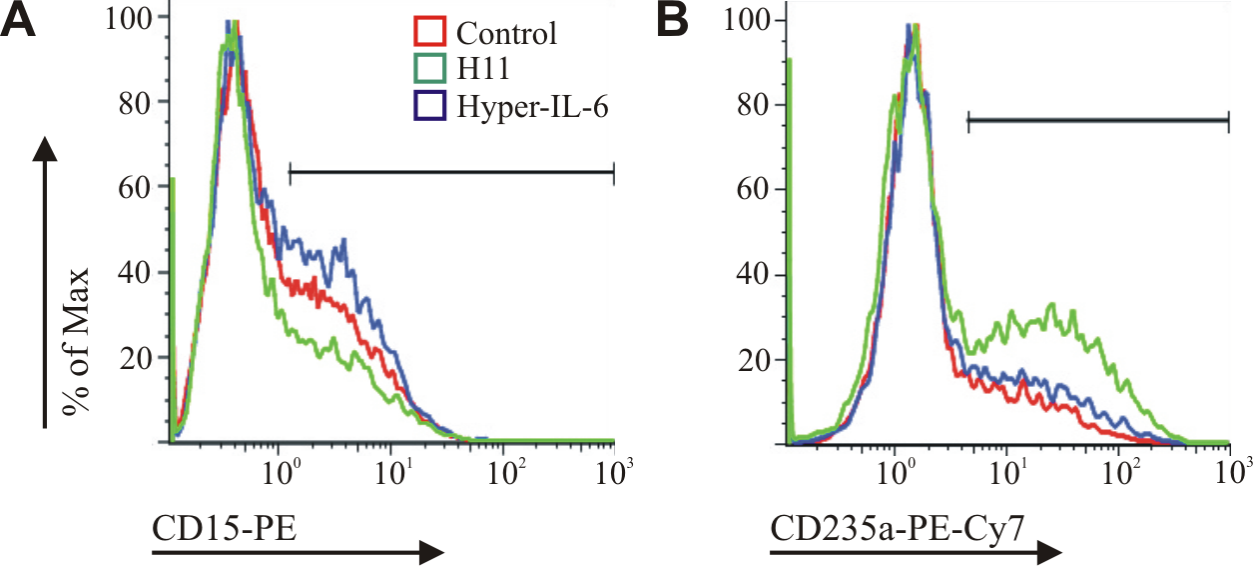
**Effect of Hyper-IL-6 and H11 on the differentiation of cord blood-derived lineage-depleted CD34+ hematopoietic progenitor cells**. Cells were seeded in X-Vivo 10 medium supplemented with rhSCF and rhIL-6 (control) and in the presence of Hyper-IL-6 or H11. After day 14 cells were harvested and analyzed by flow cytometry for the expression of CD15 (A), and CD235a (B).

To order to compare the effects of Hyper-IL-6 and H11 on differentiation of Lin-CD34+ in liquid cell cultures, analysis of expression of cell surface antigens was performed on day 14 (Figure 7). Anti-CD15 antibody was used to evaluate granulopoesis and anti-CD235a antibody to assess erythropoiesis. The number of cells expressing myeloid marker CD15 was highest in cultures containing Hyper-IL-6; (41% in Hyper-IL-6 vs. 35% in control culture and 29% in H11-Figure 7A). In contrary, H11 promoted differentiation of Lin-CD34+ towards erythroid cells. The number of CD235a+ cells was 39% in H11 stimulated culture, 27% in Hyper-IL-6 and 20% in control culture (Figure 7B).

## Discussion

Concept of the construction of recombinant fusion proteins derived from IL-6 cytokines is not new. It is based on the strategy of linking the cytokines and their cognate soluble α receptor. The α receptors posses extracellular N-terminal domain and one transmembrane domain (with exception of CNTFR, which is linked to the membrane by a lipid anchor). Structurally, following predicted regions can be distinguished: (i) signal peptide, (ii) Ig-like region-D1 domain, (iii) cytokine binding homology domain (CHD) consisting of two fibronectin-type-III-like domains (FNIII) termed D2 and D3., (iv) receptor pre-membrane region, (v) transmembrane, and (vi) cytoplasmic regions. The designed fusion proteins contained different structural parts of its α receptor. Hyper-IL-6 consisted of D2 and D3 domain of IL-6R α chain (AA residues 113-323) connected to IL-6 (AA 29-212) via artificial polypeptide linker [14]. Hyper-IL-6 is a fully active fusion protein, which mediates response at 10-1000-fold lower doses as compared to the combination of soluble IL-6 and sIL-6R molecules [14]. In analogy, another superagonist was designed named IL-11/R-FP [13]. IL-11/R-FP was created by covalently linking D2 and D3 domains of IL-11Rα (position L/109-G/318) with IL-11 (position P/29-L/199) using a 21 amino acid linker. It demonstrated 50 fold higher activity *in vitro *than the combination of IL-11 and sIL-11Rα. However, such construct was also composed of interleukin and a truncated segment of the alpha receptor (as Hyper IL-6), so it lacked naturally existing parts of the used receptor. Moreover, the used artificial linker is not a naturally occurring sequence, thus it might demonstrate potential immunogenicity when used for treatment of human patient. Hyper-CNTF was another example of a superagonist composed of sCNTFR (AA 1-346) and CNTF (AA 1-186) [15]. It differed from the previous IL-6 type fusion proteins (Hyper IL-6 and IL-11/R-FP), since it included the Ig domain of sCNTFR. Although the D3 domain of the interleukin alpha receptor is mainly involved in ligand binding [20] and its D2-D3 part is sufficient to induce biological activity *in vitro *[13,14], the role of the Ig-like domain and the membrane proximal region can not be disregarded. It was shown that the Ig-domain of the IL-6R is important for intracellular transport of IL-6R through the secretory pathway [21]. It can also be involved in interdomain stabilization or induction of conformational change of the ligand. Moreover, for fusion of CNTFR and CNTF the C-terminal end of CNTFR and N-terminal end of CNTF was used, which were linked by one additional glycine residue [15]. A synthetic polypeptide linker was not applied in order to minimize potential immunogenicity. The mentioned terminal ends are presumably flexible and sufficient to allow access of cytokine to its receptor alpha binding sites. Indeed, Hyper-CNTF was biologically active [15]. In terms of construction, our Hyper-IL-11 (H11) resembles mostly Hyper-CNTF. It is composed of full length of soluble IL-11 receptor and IL-11, and both elements are connected by natural existing parts of receptor and interleukin. It differs from Hyper-CNTF, since the additional glycine residue was not incorporated in the fusion and also the flexible termini of sIL-11Rα and IL-11 generated a longer linker. For construction of Hyper-IL-6, a 29 AA linker was used, while for IL-11/R-FP 31 AA and Hyper-CNTF 31 AA were applied [13-15]. With our hyper-molecule, a 67 AA linker was generated, since the pre-membrane region of IL-11Rα (51 amino acids) together with 16 N-terminal residues of IL-11 is not helical and presumably flexible. It should enable the proper positioning of the fusion molecule. In general, the construction of our hyper-cytokine has two major advantages: (i) its components are as close as possible to the natural forms of both proteins and (ii) omitting of the artificial linker should prevent possible immunogenicity and other side effects due to the non-natural recombinant agent.

In the case of Hyper-IL-6, the high potency of the designer cytokine is based on the fact that the affinity of IL-6 to the IL-6R is in the range of 1 nM whereas the affinity of the complex of IL-6 and IL-6R is 6R is around 10-15 pM, which is about 100 times higher. If the affinities between IL-11 and the two receptor subunits are in the same range, this would explain the high biologic activity of the H11 protein.

Although sIL-11Rα was not found in human body fluids so far, its existence has been postulated due to identification of a transcript potentially encoding sIL-11Rα [22]. The recombinant sIL-11Rα *in vitro *acts as IL-11 agonist [10-12]. In addition, the sIL-11Rα not only potentiated effects of the IL-11 on cells that are normally responsive to IL-11, but also in the presence of IL-11 it mediated a signal transduction in cells expressing gp130 molecules only [10,12]. However, the concentration of IL-11 required to mediate a biological response using sIL-11Rα had to be 10- 20-fold higher than using membrane receptor [11]. Expression of IL-11Rα is limited to certain cell types, while gp130 is present on all cells of the body. Thus, the use of the sIL-11Rα significantly widens the range of IL-11 bioactivity. Furthermore, sIL-11Rα can act as an IL-11 antagonist when tested on cells expressing the membrane IL-11Rα and gp130 [11]. The antagonism probably resulted from the competition between soluble and cellular IL-11Rα for IL-11 and/or was depended on the number of gp130 molecules [11]. Our Hyper-IL-11 acts as agonist when tested in HepG2-assay, in B9 and in Ba/F3gp130 bio-assays. Since sIL-11Rα presumably is already occupied by IL-11, it does not compete with endogenous IL-11 or membrane bound IL-11Rα, which explains its agonistic activity. The previously described molecule IL-11/R-FP also acted as agonist when tested in HepG2 and Ba/F3-gp130 assays [13]. When Ba/F3-gp130 cells were stimulated with IL-11/R-FP, a concentration 130-140 pM was sufficient for half-maximum proliferation. Our H11 induced the half-maximum proliferation at the dose of approximately 800-900 pM. However, IL-11/R-FP was non purified and its calculated concentration could be misleading. According to Pflanz et al. the concentration of IL-11/R-FP in the yeast supernatant was calculated from the band intensities of IL-11/R-FP and serial dilutions of IL-11 preparation of a known concentration measured by a Lumi-Imager [13]. Since the analyzed material was derived from not purified yeast supernatant, presumably the concentration was calculated after immunoblotting (it was not defined). Our experience indicated that antibodies anti IL-11 or IL-11Rα not necessarily recognize fusion molecule with the same affinity. When several ELISA systems were used to measure H11 concentration, different results were obtained (data not shown). The antigenic determinant at a fusion molecule may be hidden or partially hidden when compared with the epitope of parental components (IL-11 or IL-11Rα), what can explain the observed differences. In order to compare correctly the activity of both fusion proteins (H11 and IL-11/R-FP) the experiment should be conducted simultaneously using purified proteins, which concentration was calculated with the same method.

After binding to their cognate α receptor, cytokines IL-6 and IL-11 engage a homodimer of gp130 in order to elicit signal transduction. Since, both designer cytokines, H11 and Hyper-IL-6 compromise of cytokine and its cognate α receptor, they need the same receptor subunits for signal transduction. That is why H11 and Hyper-IL-6 may be functionally equivalent. According to our preliminary results H11 retains in part an IL-11Rα-specific activity that is directed by H11 interaction with gp130. In terms of hematopietic differentiation H11 increased expression of early erythroid antigen. IL-11 is well known to be involved in the expansion of megakaryiocyte progenitors, and megakariocytes derive from common megakaryocyte/erythrocyte progenitor [23]. However, Hyper IL-6 did not exert the same effect. Contrary, it stimulated myelopoiesis, what is in agreement with previous data [14]. Although, IL-6 and IL-11 share the common receptor subunit (gp130), they bind to the different sites on it [24,25], what can influence on the specific response mediated by gp130 signaling. The proliferation of TF1 cells induced by IL-11 but not by IL-6 could be inhibited by a specific antibody anti gp130 (B-P4). On the other hand, using SK-N-MC cells, the same antibody could not block the phosphorylation of gp130 induced by IL-11 and by IL-6 [24]. Moreover, PHA-activated T-cells responded to IL-6 by mobilization of a heterodimer STAT1/STAT3 and homodimer STAT1, while IL-11/sIL-11Rα by a STAT3 homodimer and STAT3/STAT1 heterodimer [10]. These results indicated that although structurally cytokines share the same signal transducing unit, its activation may elicit different effects, like different global phosphorylation level, activation of different STAT molecules what may influence the final biological activity. Such difference may be critical to activation of distinct regulation processes depending on the cell origin. However, whether this kind of differences are responsible for diverse action of H11 and Hyper-IL-6 needs to be evaluated.

Since IL-11 is a pleiotropic cytokine of hematopoietic and anti-inflammatory properties, H11 may be potentially beneficial in the treatment of various diseases. IL-11 is the only agent approved by USA Food and Drug Administration (FDA) to prevent severe thrombocytopenia and reduce the need for platelet transfusion following myelosuppressive chemotherapy for non-myeloid malignancies. Accordingly, studies of H11 in similar models are required. Even if *in vivo *studies demonstrate unacceptable toxicity it does not exclude utility of H11 for *ex vivo *expansion of megakaryocyte precursor cells. Our preliminary *in vitro *studies have indicated great potential of H11 for megakaryocyte expansion (data not shown). Moreover, recent studies have shown that IL-11 regulated the autoimmune demyelination disease-multiple sclerosis (MS) [26]. Due to its dual action: immunomodulatory and neuroprotective/regenerative, IL-11 may display a unique potential to treat MS. Loss of IL-11Rα expression was associated with severe symptoms of autoimmune encephalomyelitis (EAE) in the mouse model of MS. IL-11 treatment of mice with EAE gave partial, albeit statistically significant, therapeutic benefit. Another example of potential H11 utility is to treat infertility. Mice lacking IL-11Rα are infertile due to defective uterine response to implantation [27]. Accordingly, H11 which can omit IL-11Rα and directly achieve gp130 may prove to be beneficial in pregnancy.

## Conclusions

We constructed a designer cytokine Hyper IL-11 (H11), which is exclusively composed of naturally existing components. It contains the full length sIL-11Rα linked with the IL-11 using their natural sequences. Accordingly, H11 should not induce of the antibody production and other side effects due to the non-natural recombinant agent. The purified recombinant H11 is a fully active protein. It displayed biological activity in three different *in vitro *bio-assays: human hepatoma cell line HepG2-assay, B9 and Ba/F3-gp130 proliferation assays. Moreover, it differs in terms of differentiation of hematopoietic cells when compared to the Hyper-IL-6.

Since there are disorders due dysfunction of IL-11 or IL-11Rα, H11 may be potentially applied in the biomedical field. It may be useful for treatment of thrombocytopenia, infertility, multiple sclerosis, cardiovascular diseases or inflammatory disorders. It should be noted however, that due to the fact that Hyper-IL-11 could stimulate all cells in the body, a strategy to properly target the designer cytokine needs to be developed.

## Methods

### Cell culture

*Insect cell culture*. Sf21 (*Spodoptera frugiperda*) and High-Five BTI-TN-5B1-4 (*Trichoplusia ni*) insect cells were routinely cultured at 28°C in ambient CO_2 _in Grace's Insect Cell Culture Medium supplemented with 10% Fetal Calf Serum (FCS) and Express Five SFM medium, respectively. Monolayer cultures of Sf21 were adapted to suspension growth in Sf-900 II SFM medium on an orbital shaker platform rotating at 150 rpm. For adaptation of High-Five BTI-TN-5B1-4 cells to suspension culture, aggregated cell clamps were allowed to settle for 2 min and then samples for subculturing were taken from the upper layer of the culture. Cells were cultured in 500 ml shake flasks on an orbital shaker platform rotating at 150 rpm. All cell culture media and FCS were of Gibco Invitrogen Corporation (Grand Island, NJ) brand.

*Mammalian cell culture*. Human hepatoma cells (HepG2) were maintained in DMEM (Gibco Invitrogen, Grand Island, NJ) medium supplemented with 10% FCS. The B9 cell line, a variant of the IL-6 dependent murine hybridoma B13.29, was routinely cultured in DMEM containing 10% FCS and 10% conditioned medium from B78-IL-6 cell line (a source of IL-6)' [28]. The murine bone marrow-derived pro-B-cell line Ba/F3 stably transfected with a gp130 cDNA (Ba/F3-gp130) was maintained in DMEM medium supplemented with 10% FCS and 0.5 ng/ml mIL-3 (Sigma, St. Louis, MO). Lin-CD34+ cells were cultured in serum free X-Vivo10 culture medium (Lonza, Verviers, Belgium). All media contained 80 mg/l gentamycin (KRKA, Novo Mesto, Slowenia) and cell cultures were grown at 37°C in an atmosphere of 95% air-5% CO_2_.

### Construction of hyper IL-11 (H11)

The fragment of human IL-11 cDNA (sequence which corresponds to the amino acid residues 19-199, GenBank:ID: X58377) was amplified by a standard PCR reaction. The amplified fragment of IL-11 was cloned into the pGEM-Teasy vector (Promega, Madison, WI). The cDNA of human IL-11Rα (the amino acid residues 1-365, GenBank:ID: Z388102) was also amplified by PCR. Sequences of the used primers were as follows: IL-11 forward: 5' GCT GCT GCC CCT GGG CCA, IL-11 reverse: 5' TCC GCG GCC GCT ATG GCC GAC GTC GAC TCA CAG CCG AGT CTT CAG, IL-11Rα forward: 5' ACG CGT CGA CGC CAC CAT GGG CAG CAG CTG CTC AGG GCT G, IL-11Rα reverse: 5' AAC TCG AGG GGG GCC AGG TGG TGG CCC AGG GGC GAC AGC CTG CTC CAC AGA GTC CCT. The IL-11Rα PCR product was cloned into the pGEM-Teasy vector and then digested with *Sal*I and *Xho*I restriction enzymes. The purified fragment *Sal*I/*Xho*I was cloned into *Xho*I site of the plasmid pGEM-Teasy/IL-11 leading to the creation of the fused cDNA of IL-11Rα and IL-11 referred as Hyper IL-11 (H11). The sequence has been confirmed by DNA sequencing.

Restriction, DNA modification and amplification enzymes were supplied by Promega (Madison, WI). PCR and plasmid DNA fragments were purified using a QIAquick gel extraction kit (Qiagen, Hilden, Germany) as recommended by the manufacturer. Plasmid DNA was isolated using a QIAprep Spin miniprep Kit (Qiagen, Hilden, Germany) according to the manufacturer's protocol.

### Production of recombinant H11

For production of recombinant H11 the Bac-To-Bac Baculovirus Expression System was used (Invitrogen Corporation, Carlsbad, CA). First, the pGEM-Teasy/H11 plasmid was digested with *Sal*I/*Sph*I restriction enzymes and the purified cDNA coding for H11 was cloned into the *Sal*I/*Sph*I of pFastBac1 plasmid. The pFastBac1/H11 plasmid was transformed into DH10Bac competent cells, which contain the bacmid with a mini-*att*Tn7 target site and the helper plasmid. The mini-Tn7 element on the pFastBac1 donor plasmid was transposed to the mini-attTn7 target site on the bacmid in the presence of transposition proteins provided by the helper plasmid. The transformed cells were plated on LB-agar plates containing 50 μg/ml kanamycin, 7 μg/ml gentamycin, 10 μg/ml tetracycline, 300 μg/ml Bluo-gal, and 40 μg/ml IPTG. The selected, white colonies were grown in 3 ml clutures overnight and plasmid DNA was isolated. The obtained DNA was analyzed by PCR reaction to confirm the presence of the inserted gene. The following primers were used: Forward #9: 5' ACC CAC CCG CTA CCT CAC CT, Reverse #2: 5' GTC AGG AGC ACG GTG CT.

*Propagation the recombinant baculovirus*. The Sf21 cells were transfected with recombinant bacmid DNA using Effectene Transfection Reagent (Qiagen, Hilden, Germany) according to the manufacturer's instruction. Shortly, 8 × 10^5 ^cells were seeded into Grace's Insect Cell Culture Medium supplemented with 10% FCS in 35 mm well of a 6-well plate and incubated overnight. For transfection-complex formation, 1 μg of bacmid DNA was mixed with 8 μl of Enhancer and 25 μl of Effectene Transfection Reagent. After 10 min incubation, the Grace's Insect Cell Culture Medium supplemented with 10% FCS was added and transferred onto the cells. After 3 days, supernatant was collected and the titer of the virus was assessed using the end-point dilution method according to established procedures [29]. For amplifying viral stocks, 1.4 × 10^6 ^of Sf21 cells suspended in Sf-900 II SFM (Gibco Invitrogen, Grand Island, NJ) medium were infected at Multipicity of Infection (MOI) 0.1 for 72 h. The amplifying procedure of the recombinant H11 baculovirus was performed twice and finally the high-quality, high- titer (2.87 × 10^8 ^pfu/ml) master virus stock was obtained. The recombinant virus stock was stored at -80°C.

*Optimizing of heterologus protein production*. In order to achieve an optimal production of recombinant H11 protein, different factors, such as cell lines (Sf21 and High-Five BTI-TN-5B1-4), medium (Sf-900 II SFM and Express Five SFM), parameters of viral infection (MOI) and expression kinetics were considered. To express the recombinant gene product, suspension cultures at a cell density of 1, 1.5, and 2 × 10^6 ^cells/ml were infected at MOI 0.5, 1, 5, and 10 pfu/ml and expression of H11 was monitored by dot blot analysis at different harvest times. Optimal production of recombinant H11 protein was achieved at cell density of 1 × 10^6 ^cells/ml at MOI 5 for 48 h using High-Five BTI-TN-5B1-4 cells in Express Five SFM medium. Moreover, production of H11 was performed in the presence of Protease Inhibitor Cocktail for use in culture medium (Sigma, St. Louis, MO) at 1:1000 dilution. For dot-blot analysis, hIL-11Rα polyclonal antibody (Santa Cruz Biotechnology, Santa Cruz, CA) and goat anti rabbit IgG-HRP antibody (DakoCytomation, Glostrup, Denmark) and ECL- kit (Amersham Bioscences, Uppsala, Sweden) were used.

### Purification and gel analysis of recombinant H11 protein

Harvested supernatant was diluted with 20 mM 1,3 diaminopropane buffer pH 11.8 at the ratio of 1: 8. Next, the precipitated components were removed by centrifugation at 8 000 g/4°C and batch IEX chromatography was performed overnight at 4°C using Q Sepharose XL anion exchange bed (Amersham Bioscences, Uppsala, Sweden). The Q Sepharose XL was transferred to column and absorbed proteins were eluted using a gradually increasing gradient of salt (0-1 M NaCl) in 20 mM 1,3 diaminopropane buffer pH 10.5. Collected fractions were analyzed by SDS-PAGE gel electrophoresis and Western Blot. Proteins were separated in 10% SDS-PAGE gel and stained with Roti-Blue, Colloidal Coomassie staining (Carl Roth, Karlsruhe, Germany) according to the manufacturer's protocol. For Western blot analysis, the separated proteins were transferred to a PVDF membrane by a semi-dry electroblotting and then blocked in a solution of 10% BSA (Sigma, St. Louis, MO) in TBS-N buffer. For detection of H11, the antibodies and chemiluminescence reagent were used as mentioned before for dot blot analysis. Selected fractions containing recombinant H11 protein were pooled and concentrated by membrane filtration using membranes with a cut-off of 30 kDa (Millipore, Jaffrey, NH). PD-10 Desalting Columns (Amersham Biosciences, Uppsala, Sweden) were used for buffer exchange to PBS and H11 was sterilized by filtration using an Ultrafree-MC sterile 0,22 μm filter unit (Millipore, Jaffrey, NH). The concentration of H11 was estimated by analyzing the band intensities of H11 and serial dilution of BSA of a known concentration using Kodak Molecular Imaging Software v4.5.1.

### Stimulation of α_1 _antitrypsin in HepG2 cells by H11

5 × 10^5 ^of HepG2 cells were seeded on a 48 well plate and incubated overnight. Next day, cells were washed with serum-free DMEM and then induced with H11 in serum-free medium containing 1 μM dexamethasone. After 48 h supernatants were collected and the amounts of α_1 _antitrypsin were measured by rocket immunoelectrophoresis using antibody containing 1% agarose gels [30]. The antibody used for this study (Anti-α_1_-Antitrypsin IgG) was from Sigma (St. Louis, MO). The experiment was repeated three times in duplicate.

### Proliferation assays

The B9 cells (1 × 10^4 ^cells/well) were seeded on 96 well plate. Next, the cells were induced with recombinant hIL-11 (Sigma, St. Louis, MO) or H11 at concentrations indicated in Figure 4. The medium without stimulator was used as a negative control. The proliferation of the cells was measured after 72 h using a colorimetric assay based on the MTT reagent (Sigma, St. Louis, MO). For Ba/F3-gp130 studies, 2 × 10^4 ^cells per well were seeded on 96 well plates in complete medium and stimulated with increasing concentrations of H11. After 72 h of incubation, proliferation of Ba/F3-gp130 cells was quantified using the MTT reagent. The absorbance of the samples was measured at 562 nm with a microplate reader ELX808IV (Bio-Tek Instruments, Winooski, VT). Each experiment was carried out three times in triplicate.

### STAT3 activation in response to hIL-11 and H11

Ba/F3 and Ba/F3-gp130 cells were incubated for 2 h in serum-free medium. Next, 1 × 10^6 ^cells were stimulated with hIL-11, H11 or control medium. After 15 min incubation at 37°C, cells were fixed with Fix Buffer I (BD Biosciences Pharmingen, San Jose, CA), washed twice with staining buffer (PBS-0.1%BSA-0.1%NaN_3_) and permeabilized by adding Perm Buffer III (BD Biosciences Pharmingen, San Jose, CA) and incubating for 30 min on ice. After washing, intracellular staining was performed by adding 5 μl Alexa Fluor 647 labeled anti pSTAT3 antibody (BD Biosciences Pharmingen, San Jose, CA) or Alexa Fluor 647-IgG_2a _isotype control (BD Biosciences Pharmingen, San Jose, CA). Cells were incubated in the dark for 30 min at room temperature, washed and suspended in 0.5 ml PBS. Analysis of the cells was performed by using a BD Biosciences FACSCANTO flow cytometer and FACSDIVA software. The experiment was repeated three times.

### Propagation and differentiation of human Lin-CD34+ cells

*Isolation and culture of human Lin*-*CD34*+ *cells*. Human Lin-CD34+ cells were isolated from fresh cord blood. Light density cells were separated by Ficoll-Hypaque density centrifugation (Sigma, St. Louis, MO). Mature hematopoietic cells such as T cells, B cells, NK cells, dendritic cells, monocytes, granulocytes and erythroid cells were removed by using the Lineage Cell Depletion Kit human (Miltenyi Biotec Inc., Auburn, CA). Resulting Lin-cell fraction was enriched for CD34+ cells by using the CD34 MicroBead Kit human (Miltenyi Biotec Inc., Auburn, CA). Lin-CD34+ cells were cultured in 48-well plates (5 × 10^4 ^cells per well) in X-Vivo10 culture medium supplemented with cytokines: cocktail 50 ng/ml rhSCF (PreproTech Inc. Rocky Hill, NJ) and 20 ng/ml rhIL-6 (PreproTech Inc. Rocky Hill, NJ) as control plus 50 ng/ml Hyper-IL6 or 50 ng/ml H11. Hyper-IL-6 was constructed and produced as described previously [14,31]. Viability and cell number was counted twice weekly. Cells were cultured for two weeks.

*Flow cytometric analysis*. The differentiation of Lin-CD34+ was evaluated by flow cytometry. Cells were washed twice with PBS and stained for 30 min at 4°C with combinations of the following murine monoclonal antibodies: phycoerythrin (PE)-labeled anti-CD15 (Coulter Beckman, Marseille, France) and phycoerythrin-Cy7 (PE-Cy7)-labelled anti-CD235a (Beckman Coulter, Marseille, France). After labeling procedures, cells were washed once with PBS and analyzed using Coulter Beckman Navios flow cytometer and FlowJo software.

## Competing interests

The authors declare that they have no competing interests.

## Authors' contributions

HD-K carried out the molecular construction of H11, the optimization of the production and purification of H11, the cell proliferation study and drafted the manuscript. KG carried out the production and purification of H11 and the cell assays. EK-B carried out the propagation and differentiation study of Lin-CD34+. DI participated in the STAT 3 activation study. SR-J and AM participated in the design of the study and coordination and helped to write the manuscript. All authors read and approved the final manuscript.
